# Fowler–Nordheim Tunneling in AlGaN MIS Heterostructures with Atomically Thin *h*-BN Layer Dependence and Performance Limits

**DOI:** 10.3390/nano15151209

**Published:** 2025-08-07

**Authors:** Jiarui Zhang, Yikun Li, Shijun Luo, Yan Zhang, Man Luo, Hailu Wang, Chenhui Yu

**Affiliations:** 1 National Key Laboratory of Infrared Detection Technologies, School of Microelectronics and Integrated Circuits (Jiangsu Key Laboratory of Semi. Dev. & IC Design, Package and Test), Nantong University, Nantong 226019, China; 2 State Key Laboratory of Infrared Physics, Shanghai Institute of Technical Physics, Chinese Academy of Sciences, Shanghai 200083, China

**Keywords:** few-layer *h*-BN, Fowler–Nordheim tunneling effect, AlGaN-based MIS, TCAD simulation, electrical characteristics

## Abstract

Hexagonal Boron Nitride (*h*-BN) is an exceptional dielectric material with significant potential for high-performance electronic and optoelectronic devices. While previous studies have explored its role in GaN-based MIS (metal/insulator/semiconductor) structures, the influence of few-layer *h*-BN on AlGaN MIS devices—particularly with varying Al compositions—remains unexplored. In this work, we systematically investigate the Fowler–Nordheim tunneling effect in few-layer *h*-BN integrated into AlGaN MIS architectures, focusing on the critical roles *h*-BN layer count, AlGaN alloy composition, and interfacial properties in determining device performance. Through combined simulations and experiments, we accurately determine key physical parameters, such as the layer-dependent effective mass and band alignment, and analyze their role in optimizing MIS device characteristics. Our findings reveal that the 2D *h*-BN insulating layer not only enhances breakdown voltage and reduces leakage current but also mitigates interfacial defects and Shockley–Read–Hall recombination, enabling high-performance AlGaN MIS devices under elevated voltage and power conditions. This study provides fundamental insights into *h*-BN-based AlGaN MIS structures and advances their applications in next-generation high-power and high-frequency electronics.

## 1. Introduction

Few-layer hexagonal boron nitride (*h*-BN) serves as an excellent two-dimensional (2D) insulating material due to its exceptional dielectric characteristics. Its atomically flat and chemically inert surface effectively mitigates interfacial electron scattering, which boosts the mobility of graphene to 3.7 × 10^4^ cm^2^ V^−1^ s^−1^ [1] and enhances the threshold voltage stability in MoS_2_ transistors [2]. With a wide bandgap of about 6 eV, *h*-BN establishes a large insulating barrier of over 3 eV. This barrier can be traversed by electrons under a high electric field through the Fowler–Nordheim (F–N) tunneling mechanism, a property that significantly optimizes the rectification characteristics of heterojunctions like the black phosphorus/*h*-BN/rhenium diselenide (BP/*h*-BN/ReSe_2_) structure [3,4]. Moreover, the pronounced dielectric anisotropy of *h*-BN supports the confinement of hyperbolic phonon polaritons (HPPs) [5], enabling graphene/*h*-BN detectors to achieve a high room-temperature sensitivity of 82 pW/Hz^1/2^ [6]. In addition, *h*-BN features excellent thermal conductivity (>400 W·m^−1^·K^−1^) [7], which allows for effective heat management in devices and underpins the development of 20 ns ultrafast non-volatile memory [8] and multi-functional integrated devices [9]. Therefore, an in-depth analysis of the electrical properties of *h*-BN is a critical cornerstone for advancing emerging technologies such as flexible electronics, quantum light sources, and high-speed memory.

Wide bandgap semiconductors, particularly III-nitrides, are foundational for next-generation electronics. Their excellent properties are expanding the performance and functionality frontiers of electronic devices [10,11,12,13]. Echoing the value of research into the electrical properties of few-layer *h*-BN, an in-depth investigation of gallium nitride (GaN) metal–insulator–semiconductor (MIS) structures is equally crucial for realizing high-performance power electronic devices. For instance, Whiteside et al. demonstrated that employing vertically ordered *h*-BN as a gate insulator in MIS-HEMTs significantly reduces gate leakage current compared to conventional Schottky gate electrodes, attributing the improved performance to the high-quality heterojunction interface [14]. As the fundamental building block of high-voltage optoelectronic devices, GaN MIS structures have long been plagued by the high density of interface states (up to 10^14^ cm^−2^ eV^−1^) at the interface with conventional insulators, leading to severe carrier recombination loss [15,16]. By introducing few-layer *h*-BN as the insulating layer, its atomically smooth surface (with a defect density of only 4.3 × 10^11^ cm^−2^ eV^−1^) [17] significantly suppresses interfacial state scattering. This reduces the SRH recombination current to a negligible level, thereby enhancing carrier transport efficiency [18]. Laleyan et al. leveraged the p-type properties of boron vacancies in *h*-BN to realize the first Mg-dopant-free Al(Ga)N/*h*-BN nanowire LEDs, which exhibited high output power and electrical efficiency [19]. Concurrently, under high voltage bias, the F–N tunneling characteristic of *h*-BN dominates the conduction mechanism, enabling a high threshold voltage of 4.28 V and a large on-current of 21.2 mA. This results in a power figure of merit of 25.8 mW, an improvement of over 40% compared to conventional structures [20,21]. This design also yields an ultra-low dark current (<10^−11^ A), and in parallel, the intrinsic wide-bandgap nature of *h*-BN has paved a new avenue for high-performance deep-ultraviolet photodetectors [22,23]. For instance, Lu et al. fabricated graphene/*h*-BN/GaN heterostructure photodetectors where the *h*-BN interlayer increased the potential barrier, thereby reducing dark current and enhancing the device’s on/off ratio [24].

Aluminum (Al) doping optimizes device performance by synergistically modulating the energy band structure, charge carriers, and interface properties of the GaN MIS structure. Increasing the Al composition to 15% widens the bandgap by approximately 0.3 eV, which enhances the Schottky barrier to suppress leakage current [25,26]. However, excessive Al composition (>20%) induces interface defects due to lattice mismatch, leading to a density as high as 10^14^ cm^−2^ eV^−1^ [17]. The minimum contact resistance is achieved at a doping concentration of 3 × 10^18^ cm^−3^, whereas concentrations exceeding 5 × 10^18^ cm^−3^ cause a mobility reduction of over 30% due to increased ionized impurity scattering. Furthermore, Al doping induces the formation of an Al-Ni alloy at the Ni/Au electrode, increasing the work function (WF) by 0.3–0.5 eV to optimize band alignment [27]. Experiments have demonstrated that a combination of 15% Al composition and a 3 × 10^18^ cm^−3^ doping concentration improves threshold stability by 40% and achieves a power figure of merit of 28.6 mW [9]. However, the potentially dominant role of interface traps at the graded doping layer in determining dark current warrants attention. In HgCdTe n-B-n detectors, for example, such traps increase the dark current by one to two orders of magnitude [28]. In contrast, *h*-BN can effectively suppress this phenomenon, owing to its atomically flat surface with a low interface state density (4.3 × 10^11^ cm^−2^ eV^−1^) [18].

To contextualize our contribution, it is instructive to survey the landscape of MIS structures (Table 1). While conventional 3D insulators like Al_2_O_3_ have been extensively studied [21,29,30,31,32,33], the integration of 2D insulators, particularly *h*-BN, represents a paradigm shift [34,35]. Prior works have already demonstrated the potential of *h*-BN with conventional Si and 2D semiconductors like MoS_2_, leveraging its atomically smooth surface to reduce scattering and improve mobility [18,20,36].

Significant progress in fabrication techniques is now making the integration of *h*-BN with III-nitride semiconductors practically feasible. While various methods exist, metalorganic chemical vapor deposition (MOCVD) has emerged as a particularly compelling approach for scalable manufacturing [37,38,39]. As a key fabrication method for AlGaN devices, MOCVD’s demonstrated capability for wafer-scale growth of *h*-BN layers offers a seamless integration pathway. Seminal works by Majety et al. have established the viability of this approach [40]. More recently, the refinement of MOCVD techniques, such as pulsed-mode growth, has enabled the direct synthesis of atomically thin *h*-BN layers on AlGaN [41]. The technological maturity of MOCVD thus provides a solid foundation for the mass production of advanced *h*-BN/AlGaN heterostructures, paving the way for their widespread adoption in next-generation electronics. Building on this foundation, our work focuses on the critical and promising combination of *h*-BN with a wide-bandgap AlGaN semiconductor. As summarized in Table 2, this system offers fundamental advantages over its conventional Al_2_O_3_-based counterpart. The transition from a chemically bonded Al_2_O_3_/AlGaN interface [42] to a van der Waals physisorbed *h*-BN/AlGaN interface [38] is a pivotal change that fundamentally alters the device physics. This shift inherently suppresses the formation of interface traps—a primary bottleneck that plagues the dynamic performance of conventional MIS structures. Meanwhile, the dominant carrier transport mechanism evolves from a complex, trap-assisted process in Al_2_O_3_-based devices to a more predictable, intrinsic Fowler–Nordheim tunneling in *h*-BN-based systems [42,43,44]. Furthermore, the superior in-plane thermal conductivity of *h*-BN provides a crucial thermal management pathway absent in thermally resistive Al_2_O_3_ [38,45].

Although prior research has confirmed the immense potential of *h*-BN in GaN-based devices, the complex synergistic and constraining relationships among the multi-dimensional parameters in MIS structures with an AlGaN semiconductor layer have not been systematically elucidated. As a result, their optimal design lacks clear theoretical guidance. To clarify this complex interplay and establish definitive design criteria, this work systematically investigates the comprehensive impact of *h*-BN thickness, AlGaN composition, doping concentration, and metal WF on device performance using Sentaurus TCAD numerical simulations. The findings reveal that device performance is governed by the interplay between the thickness-dependent tunneling barrier of *h*-BN and the intrinsic properties of the AlGaN semiconductor layer, such as its energy band structure and carrier concentration. Furthermore, owing to the nearly ideal van der Waals interface formed between *h*-BN and AlGaN, the impact of non-ideal effects, such as interface traps and SRH recombination, on device performance is considered negligible. To provide a holistic performance benchmark, a Figure of Merit (FOM) is introduced. We conduct a multi-parameter sensitivity analysis and systematically map the maximum achievable FOM by co-optimizing key electrical parameters for various structural combinations. In addition, we assess the device’s thermal characteristics to confirm that the derived design guidelines remain robust and applicable within a practical operational temperature window. This work yields critical physical insights and clear design guidelines for developing high-performance AlGaN/*h*-BN MIS electronic devices.

## 2. Materials and Methods

In this work, we numerically simulated the electrical properties of the MIS structure utilizing the SDevice module within the Sentaurus TCAD suite. Our simulation framework self-consistently solves a set of fundamental equations, including the Poisson equation, the electron and hole continuity equations, and the carrier transport equations, which together dictate the carrier transport phenomena in the semiconductor. This methodology enables the accurate computation of carrier movement and the resulting electrical characteristics.

**Figure 1:**
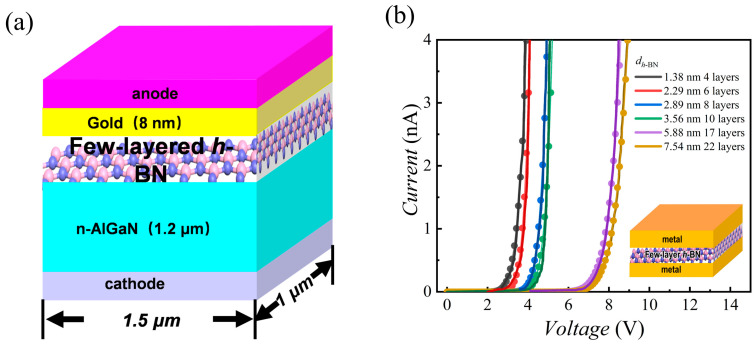
(**a**) Schematic structure of the AlGaN-based MIS blocks with few-layered *h*-BN; (**b**) Current–voltage (I–V) characteristics for devices with varying *h*-BN thicknesses, showing a comparison between simulated results (solid lines) and experimental data (dots). The inset illustrates the metal/*h*-BN/metal structure used for model validation. The thickness of *h*-BN corresponds to the number of layers: 1.38 nm corresponds to 4 layers, 2.29 nm to 6 layers, 2.89 nm to 8 layers, 3.56 nm to 10 layers, 5.88 nm to 17 layers, and 7.54 nm to 22 layers.

The proposed device is a vertical AlGaN-based MIS structure, as shown in Figure 1a. It features several atomic layers of *h*-BN as a tunneling insulator stacked on a 1.2 µm n-AlGaN layer, with an 8 nm gold anode. The device’s lateral dimensions are 1.5 µm × 1 µm. The study focuses on the combined impact of tunneling barrier width and semiconductor band structure on the electrical characteristics, systematically explored by jointly varying the *h*-BN thickness (number of layers) and the Al composition in AlGaN.

Multiple studies have confirmed that when a thin *h*-BN layer is used as the tunneling layer, carrier transport is predominantly governed by the F–N tunneling mechanism [46].

Based on this theory, we perform an in-depth analysis of the influence of thickness-dependent tunneling behavior on the electrical characteristics of metal–insulator–semiconductor (MIS) structures that incorporate a thin *h*-BN layer as the insulator.

The theoretical expression for F–N tunneling is given by [18]:

(1) $$J_{F-N}=AF_{ins}^{2}exp(-\frac{B}{F_{ins}})$$

(2) $$A=\frac{q^{3}m}{8\pi h\varphi_{B}m^{*}}$$

(3) $$B=\frac{8\pi \sqrt{2m^{*}}\varphi_{B}^{\frac{3}{2}}}{3hq}$$

Here, *J*_F-N_ is the tunneling current density and *F*_ins_ is the electric field across the insulator, while the coefficients *A* and *B* are physical parameters reflecting the influence of the *h*-BN’s electron effective mass (m*) and interfacial barrier height (*φ*_B_), where *h* is Planck’s constant and *q* is the elementary charge.

The fundamental parameters for AlGaN used in our simulation are summarized in Table 3. These values are based on or calibrated against previously reported experimental and theoretical data.

The carrier recombination mechanism significantly affects the carrier concentration distribution and its dynamics. The introduction of an *h*-BN interlayer in the gold/AlGaN structure may introduce a substantial number of deep-level defects in the AlGaN surface region. The SRH recombination model describes the process of carrier recombination via these deep-level traps within the bandgap. The introduction of such levels is expected to markedly alter the SRH recombination rate ($R_{net}^{SRH}$) in AlGaN. As SRH recombination is a key physical mechanism that governs carrier distribution and ultimately determines the performance of devices, a thorough investigation into its effect on device performance is crucial. The physical formula for calculating $R_{net}^{SRH}$ is:

(4) $$R_{net}^{SRH}=\frac{np-n_{i}^{2}}{\tau_{p}\left(n+n_{i}exp\left(\frac{E_{trap}}{kT}\right)\right)+\tau_{n}\left(p+n_{i}exp\left(\frac{{-E}_{trap}}{kT}\right)\right)}$$

where τ*_p_* and τ*_n_* denote the hole and electron lifetimes, respectively. The term *E*_trap_, representing the energy offset between the intrinsic Fermi level and the trap level, is set to zero under ideal conditions. This places the trap level at mid-gap, which maximizes the carrier recombination probability.

The high concentration of dopants introduces several second-order effects, including bandgap narrowing that occurs when impurity states merge with the band edges, a phenomenon well described by the Jain–Roulston model. Moreover, increased impurity scattering degrades carrier mobility, an effect quantitatively modeled by the Masetti mobility formulation.

The intrinsic physical properties of *h*-BN, such as its bandgap and dielectric constant, are known to be thickness-dependent. As a critical parameter for F–N tunneling, the layer-dependent effective mass (m*) was precisely extracted based on prior research [53], allowing us to build an accurate tunneling model.

To validate this model, we simulated a metal/*h*-BN/metal structure and compared the results with experimental data. As the work function of gold is about 5 eV and the affinity energy of *h*-BN is 2 eV, the barrier height (*φ*_B_) can be determined. Thereafter, the physical parameters, *A* and *B*, can be derived. As shown in Figure 1b, the excellent agreement between our theoretical curves and the experimental measurements [54] across a range of *h*-BN thicknesses confirms the general validity of our F–N model for *h*-BN as a tunnel barrier.

## 3. Results and Discussion

**Figure 2:**
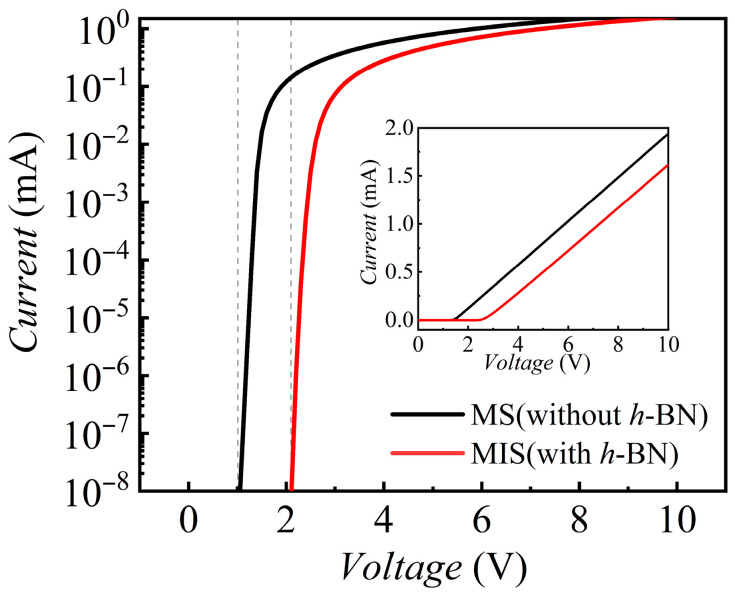
I–V characteristics comparing a conventional Au/n-AlGaN Schottky (MS) diode (black curve) with the Au/*h*-BN/n-AlGaN MIS counterpart (red curve).

Figure 2 illustrates the effect of inserting a few layers of *h*-BN at the gold/n-AlGaN interface on the device’s electrical characteristics, by comparing the current–voltage (*I*–*V*) curves of a conventional Metal-Semiconductor (MS) Schottky structure and a MIS structure.

It is clearly observed from the figure that the introduction of the *h*-BN insulating layer significantly alters the turn-on characteristics of the device. As shown, compared to the MS device, the *V*_th_ (at which the current reaches 1 × 10^−11^ A) [55] of the MIS device is markedly increased, rising from approximately 1.0 V to about 2.1 V.

**Figure 3:**
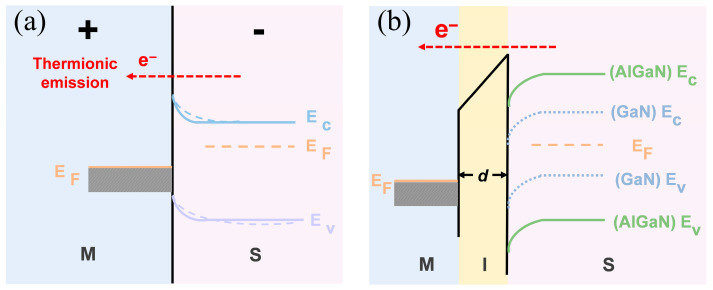
Energy-band diagrams under a large positive bias. (**a**) Gold/n-AlGaN(GaN) MS contact showing electron transport across the Schottky barrier. (**b**) Gold/*h*-BN/n-AlGaN(GaN) MIS contact illustrating the additional tunneling barrier introduced by the few-layer *h*-BN.

Figure 3 intuitively elucidates the fundamental difference in carrier transport mechanisms between the MS and MIS structures through their energy band diagrams, thereby explaining the disparity in their electrical characteristics.

In a conventional MS Schottky structure, as shown in Figure 3a, the application of a forward bias causes the energy bands on the semiconductor side to bend downward, effectively lowering the Schottky barrier height. At this point, carrier transport is primarily governed by thermionic emission, wherein electrons overcome the reduced barrier using thermal energy to form the on-state current. Due to the relatively low barrier, the device can be turned on at a lower voltage, thus exhibiting a lower *V*_th_. In contrast, for the MIS structure incorporating an *h*-BN insulating layer, as depicted in Figure 3b, the dominant transport mechanism fundamentally shifts to quantum tunneling. The *h*-BN layer establishes a high and wide potential barrier. When a forward bias is applied, electrons must tunnel through this triangular barrier, which is tilted by the *F*_ins_. This transport mechanism through a triangular barrier under a high *F*_ins_ is known as F–N tunneling [53,56]. Because the tunneling probability is highly sensitive to the barrier’s thickness and height, a higher voltage is required to achieve conduction, which explains why the MIS structure exhibits a higher *V*_th_.

The Al composition in Al_x_Ga_1−x_N is a critical design parameter in heterostructure engineering, dictating the material’s band structure and polarization for the optimization of key device metrics [57,58]. Figure 3b illustrates the effect of the Al composition in AlGaN on the energy band structure. Due to its smaller electron affinity compared to GaN, AlGaN has a relatively higher conduction band minimum. This results in a higher initial energy state for electrons in the AlGaN, which in turn effectively reduces the height of the tunneling barrier electrons need to overcome. Therefore, by tuning the Al composition, the tunneling probability can be effectively modulated. This provides another critical degree of freedom, in addition to the *h*-BN thickness, for the fine-tuned design of the device’s *V*_th_ and on-state resistance.

**Figure 4:**
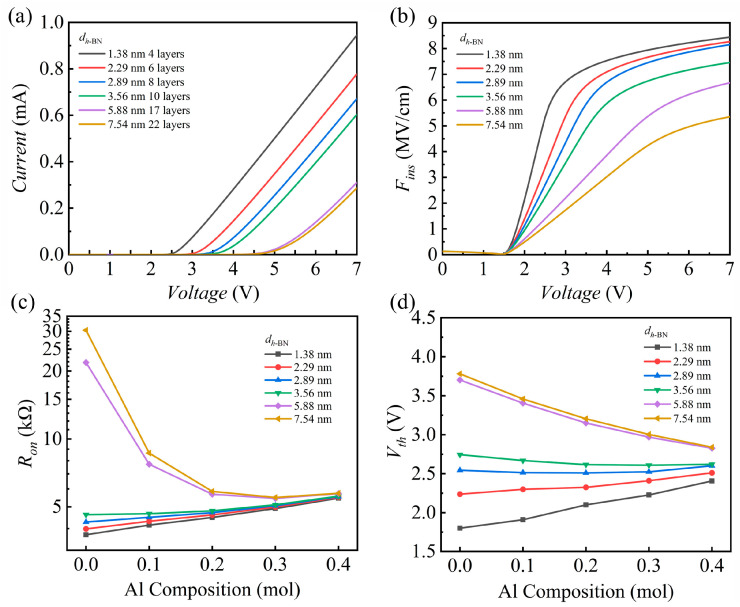
(**a**) *I*–*V* curves of the GaN-based MIS blocks for several thicknesses of *h*-BN. (**b**) Variations of *F_ins_* for several thicknesses of *h*-BN. (Al Composition = 0.2, *doping* = 1 × 10^16^ cm^−3^) (**c**) *R*_on_ and (**d**) *V*_th_ versus Al composition for several thicknesses of *h*-BN.

Since the device performance is fundamentally governed by F–N tunneling, the thickness of the *h*-BN insulating layer emerges as a critical design parameter that directly governs the tunneling probability, thus ultimately dictating the device’s electrical performance. Therefore, a detailed analysis of this layer-dependence is crucial. Figure 4 presents a systematic investigation into the effects of the *h*-BN thickness (*d_h_*_-BN_) and the Al composition in the AlGaN channel layer on the electrical characteristics of the MIS device. Figure 4a–d illustrates the evolution of the device’s current–voltage (*I–V*) characteristics, *F_ins_*, *R*_on_ (magnitude of the structure’s on-state resistance using the slope of the well-linearized region in the *I*–*V* curve), and *V*_th_, respectively.

At a fixed Al composition, the device’s electrical characteristics exhibit a clear dependence on the insulator thickness. As shown in Figure 4a, *V*_th_ increases significantly with increasing *d_h_*_-BN_, while the corresponding *I*_on_ (on state current under a positive bias of 7 V) decreases [59]. This result is in excellent agreement with the F–N tunneling theory, described by Equation (1), which posits that the carrier tunneling probability is extremely sensitive to the insulator thickness and *F*_ins_. A thicker *h*-BN layer sustains a lower *F*_ins_ under the same applied bias, as depicted in Figure 4b. This drastically suppresses the carrier tunneling efficiency, thereby leading to a higher *V*_th_ and *R*_on_.

When the Al composition in the AlGaN layer is varied, the electrical characteristics display a more complex evolutionary trend that is correlated with *d_h_*_-BN_. As seen in Figure 4d, for thin *h*-BN layers like 1.38 nm and 2.29 nm, *V*_th_ increases monotonically with the Al composition. At an intermediate thickness like *d_h_*_-BN_ = 3.56 nm, *V*_th_ exhibits a transitional behavior, first increasing and then decreasing. When the *h*-BN layer is further thickened to 5.88 nm and beyond, *V*_th_ instead decreases monotonically as Al composition increases. *R*_on_, shown in Figure 4c, displays a similar dependence: under thin *h*-BN conditions, *R*_on_ increases with Al composition, whereas for thick *h*-BN, it shows a trend of first decreasing and then increasing.

On one hand, a higher Al composition lowers the electron affinity of the AlGaN material, which effectively reduces the tunneling barrier height at the *h*-BN/AlGaN interface. The F–N tunneling formula dictates that a lower barrier leads to a substantially higher tunneling probability. Consequently, this mechanism tends to decrease both *V*_th_ and *R*_on_.

On the other hand, increasing the Al composition elevates the bulk resistivity of the AlGaN semiconductor layer, attributable to the lower carrier mobility of AlN compared to GaN. When a constant total bias is applied, a greater voltage drop occurs across the more resistive AlGaN layer. This diminishes the effective voltage across the *h*-BN insulator and the resultant *F*_ins_. The suppression of the tunneling process by this weakened field is a mechanism that tends to raise *V*_th_ and *R*_on_.

Therefore, the ultimate electrical behavior of the device depends on the relative dominance of these two opposing effects. In devices with a thin *h*-BN layer, where tunneling is relatively facile, the field reduction caused by the higher AlGaN resistivity dominates. This manifests as an increase in both *V*_th_ and *R*_on_ with rising Al composition. Conversely, for thick *h*-BN devices, the tunneling process is inherently limited. Here, the reduction in the tunneling barrier height becomes the crucial factor. The positive impact of this barrier reduction surpasses the negative effect of the diminished field, leading to a reduction in *V*_th_ and an optimized *R*_on_.

**Figure 5:**
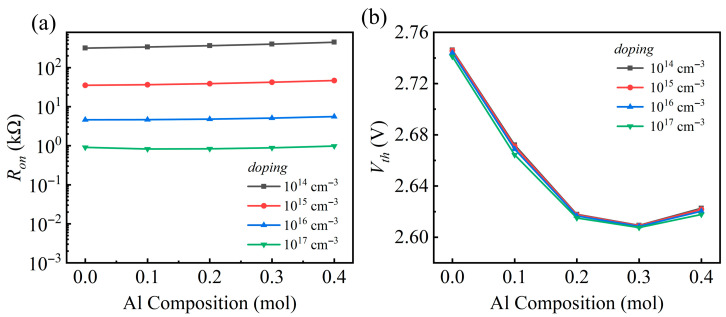
(**a**) *R*_on_ and (**b**) *V*_th_ as functions of Al composition for doping levels ranging from 10^14^ to 10^17^ cm^−3^.

The doping concentration in the AlGaN layer directly determines the carrier density at the *h*-BN interface, which in turn modulates the F–N tunneling efficiency through the barrier. A systematic study of the effects of AlGaN doping concentration (ranging from 10^14^ to 10^17^ cm^−3^) and Al composition on the device’s electrical characteristics was conducted, with GaN (Al composition = 0) as the reference. Figure 5a,b shows the *R*_on_ and *V*_th_ as a function of the Al composition. The analysis reveals that at a constant Al composition, the device’s electrical properties respond differently to the doping concentration. *R*_on_ drops by several orders of magnitude as doping increases (Figure 5a). This is attributed to a larger supply of electrons for F–N tunneling at higher doping levels post turn-on, which facilitates a steeper current rise and hence a lower *R*_on_. Conversely, *V*_th_ exhibits negligible sensitivity to the doping concentration (Figure 5b), as it is predominantly governed by the potential barrier at the AlGaN/*h*-BN interface, whose height shows little dependence on doping.

When viewed as a function of Al composition, the *V*_th_ trend is remarkably consistent across all doping levels, initially falling with increasing Al composition to a minimum around a 0.3 mole fraction before rising slightly, reaffirming its weak doping dependence. In contrast, the behavior of the *R*_on_ is contingent on the doping level. For concentrations ranging from 10^14^ to 10^16^ cm^−3^, *R*_on_ is observed to rise monotonically with the Al composition. At a high doping level of 10^17^ cm^−3^, this trend reverses, with *R*_on_ displaying a non-monotonic behavior of first decreasing and then increasing. This occurs because changes in Al composition exert two opposing influences on the tunneling process, giving rise to a competition mechanism. Altering the doping concentration will affect the balance of this competition. Consequently, *R*_on_ exhibits the observed trend as the Al composition changes.

**Figure 6:**
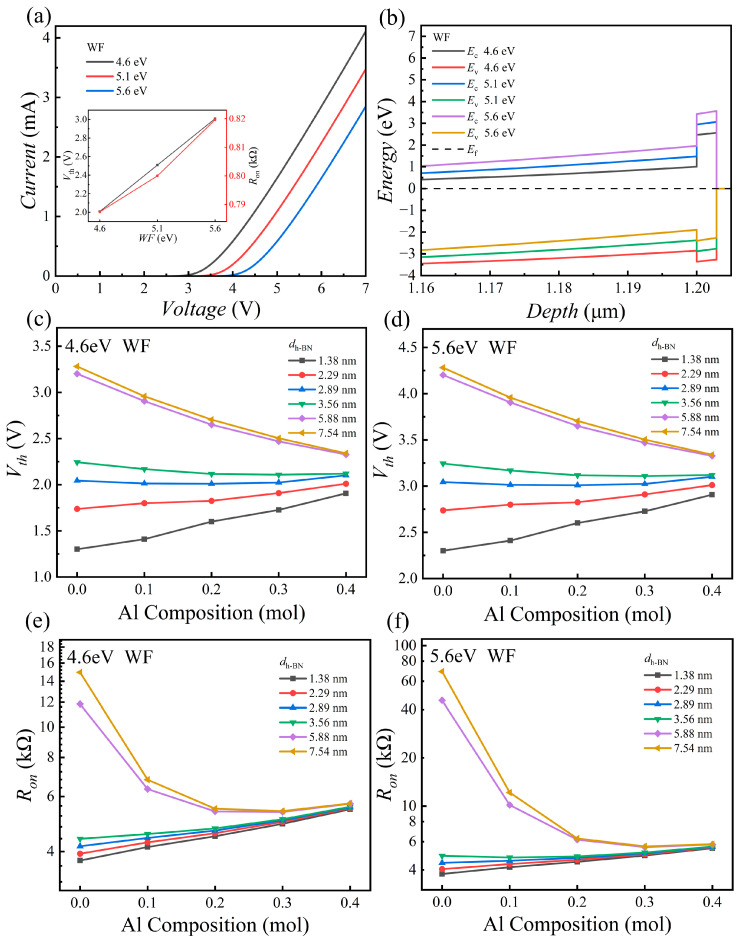
Influence of contact-metal WF and *h*-BN thickness. (**a**) *I*–*V* curves with different contact metals in AlGaN-based MIS blocks, with an inset showing the trend of *V*_th_ and *R*_on_; (**b**) influence of metal WF on energy band structure without applied bias voltage (*d_h_*_-BN_ = 2.89 nm, *doping* = 1 × 10^17^ cm^−3^); (**c**,**d**) *V*_th_ versus Al composition and *h*-BN thickness for W (4.6 eV) and Pt (5.6 eV) contacts, respectively; (**e**,**f**) *R*_on_ versus Al composition and *h*-BN thickness for W and Pt contacts, respectively (*doping* = 1 × 10^16^ cm^−3^).

The work function (WF) of the contact metal establishes the barrier height at the *h*-BN interface (Figure 3b), which in turn critically dictates the electrical properties of the MIS device. To investigate the modulating effect of the metal WF on the electrical characteristics of the MIS device, this study introduces platinum (Pt, WF = 5.6 eV) as a high-work-function material and tungsten (W, WF = 4.6 eV) as a low-work-function material for a comparative analysis, using gold (Au, WF = 5.1 eV) as the baseline. Figure 6a illustrates that for a fixed device configuration (*d_h_*_-BN_ = 2.89 nm, Al Composition = 0.2), the metal WF markedly influences the *I*–*V* characteristics. A larger WF simultaneously raises *V*_th_ and *R*_on_, thereby lowering *I*_on_.

Figure 6b explains this behavior through the corresponding energy-band diagram. A higher metal WF increases the potential barrier at the *h*-BN/AlGaN heterointerface, thus making elect *R*_on_ tunneling more difficult. Consequently, a greater forward bias is necessary to activate the device, leading to a reduced *I*_on_. Figure 6c–f further illustrates the evolution of *V*_th_ and *R*_on_ as a function of Al Composition and *h*-BN thickness under different work functions. Although the high-work-function metal (5.6 eV) systematically results in higher baseline values for *V*_th_ and *R*_on_ (compare Figure 6c with Figure 6d, and Figure 6e with Figure 6f), the relative trends of their variation with Al Composition and *h*-BN thickness remain highly consistent with those of the low-work-function metal (4.6 eV). For example, the non-monotonic dependence of *V*_th_ and *R*_on_ on the Al Composition is preserved regardless of WF.

However, it is noteworthy that the impact of WF is amplified under specific conditions. Particularly when the Al composition is low and the *h*-BN is thick (corresponding to a high initial resistance state), using a high WF metal leads to a dramatic increase in *R*_on_, as shown in Figure 6f. This indicates that when the tunneling current is already limited by a thicker barrier layer, the additional barrier increment introduced by the high WF exerts a more drastic impact on the device’s on-resistance.

To evaluate the impact of the *h*-BN/AlGaN interface quality on device performance, the effects of the interface trap density (Dit) and SRH recombination on the device’s electrical characteristics were systematically investigated in this study.

*h*-BN has excellent properties of an atomically flat surface and an absence of dangling bonds, which in turn enable the formation of a near-ideal van der Waals heterointerface with significantly reduced defects when integrated with III-nitrides [17].

**Figure 7:**
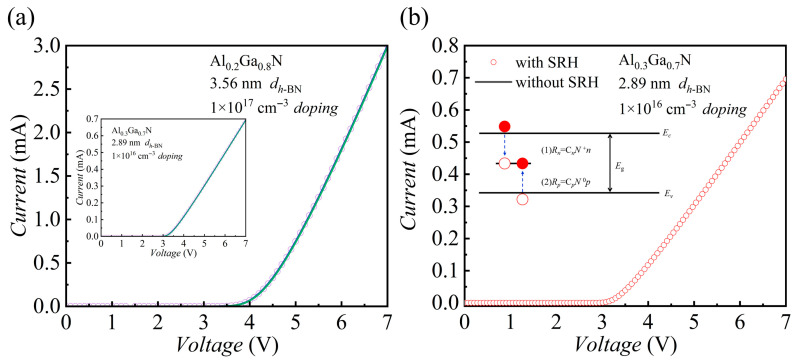
(**a**) *I–V* curves for AlGaN-based MIS blocks with interface-trap densities from 0 to 5 × 10^12^ cm^−2^ eV^−1^ at the *h*-BN/AlGaN interface. (**b**) *I–V* comparison with (open circles) and without (solid line) SRH recombination; inset schematically depicts electron and hole capture processes within the bandgap.

Figure 7a displays the impact of Dit on the *I*–*V* characteristics. In this analysis, we varied Dit from an ideal zero up to 5 × 10^12^ cm^−2^ eV^−1^ for two distinct MIS device structures. Remarkably, the *I*–*V* curves show virtually no deviation across this entire range, indicating that the high-quality *h*-BN interface substantially suppresses the influence of interface traps on carrier transport, even at high densities.

Building on this finding, we further investigated the bulk SRH recombination mechanism [60]. Figure 7b compares the *I*–*V* curves simulated with and without the inclusion of the SRH recombination model. The perfect overlap of the two curves confirms that in the gold/*h*-BN/AlGaN structure, *I*_on_ is governed entirely by the tunneling mechanism, and the contribution from SRH recombination current is negligible. The underlying physical reason is that when the device is under positive bias, electrons accumulate at the interface on the n-AlGaN side; however, due to the extremely low hole concentration, effective electron–hole recombination is unlikely to occur (as illustrated in the inset of Figure 7b). According to the SRH recombination theory, as shown in Equation (4), the scarcity of minority carriers drives the SRH recombination rate toward zero, effectively eliminating this current path.

In summary, the insertion of *h*-BN insulating layer drastically improves the interface quality. Consequently, device performance is governed by intrinsic tunneling processes, as the impact of interface defects and recombination processes becomes negligible.

**Figure 8:**
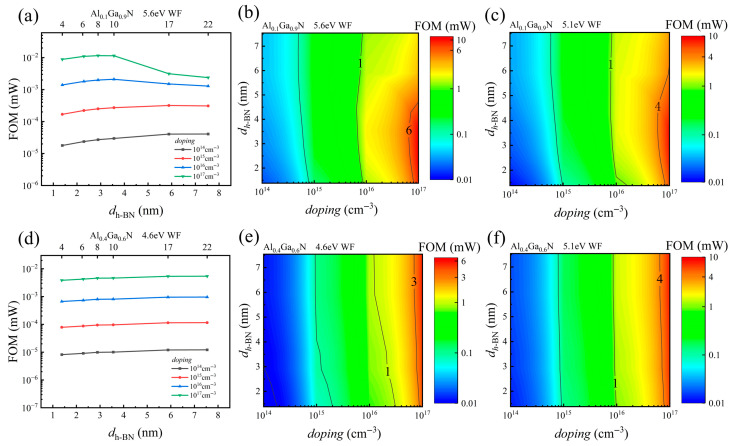
(**a**,**d**) Line plots of FOM versus *h*-BN thickness for several doping concentrations; (**b**,**c**,**e**,**f**) contour maps of FOM versus *h*-BN thickness and doping concentration. The specific conditions for each subplot are (**a**,**b**) Al_0_._1_Ga_0.9_N, 5.6 eV WF; (**c**) Al_0.1_Ga_0.9_N, 5.1 eV WF; (**d**,**e**) Al_0.4_Ga_0.6_N, 4.6 eV WF; (**f**) Al_0.4_Ga_0.6_N, 5.1 eV WF.

For a holistic assessment and optimization of the device’s performance, especially to navigate the trade-off between *V*_th_ and *R*_on_, we introduce the Figure of Merit (FOM), defined as FOM = *V*_th_^2^/*R*_on_ [61]. This FOM simultaneously accounts for the device’s threshold voltage and the rate of current increase during device turn-on, making it a critical benchmark for overall performance evaluation. Examining how the FOM varies with *h*-BN thickness, AlGaN doping, Al Composition, and metal WF yields clear design guidelines. Figure 8a,d reveals two distinct trends in the FOM versus *h*-BN thickness. In the first, the FOM peaks and then falls as the barrier thickens. This behavior is observed, shown in Figure 8a, in devices featuring a high WF metal (5.6 eV WF) and a low-Al-composition AlGaN (Al_0.1_Ga_0.9_N), particularly at higher doping concentrations (≥10^16^ cm^−3^). The second trend, shown in Figure 8d, is a monotonic rise in FOM over the whole thickness range. This occurs when a low WF metal (4.6 eV WF) is paired with a high-Al-composition AlGaN (Al_0.4_Ga_0.6_N).

The 2D contour maps in Figure 8b,c,e,f offer an overall view of the FOM distribution in various parameter spaces, fully corroborating the 1D plot results. This divergence in behavior is rooted in the competing growth rates of *V*_th_ and *R*_on_ as a function of *h*-BN thickness. Although both quantities rise monotonically, R_on_ responds more strongly under a high WF, low-Al AlGaN, and heavy doping, so its growth eventually exceeds that of *V*_th_^2^. Consequently, for thin *h*-BN, the *V*_th_^2^ term’s growth prevails, boosting the FOM; however, beyond a critical thickness, the escalating *R*_on_ dominates, causing the FOM to decline. In the opposite scenario, the growth of *V*_th_^2^ consistently outstrips or matches that of *R*_on_, leading to a monotonically increasing FOM trend. These insights establish a theoretical foundation for synergistically optimizing multi-dimensional parameters to achieve maximum FOM tailored to specific applications.

**Figure 9:**
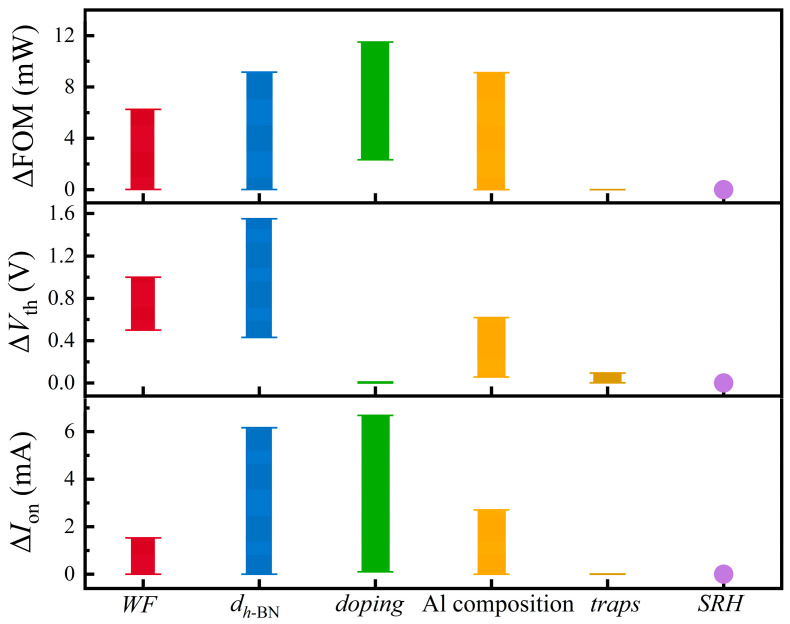
Scope of the effect of each physical parameter on FOM, *V*_th_, and *I*_on_ in AlGaN-based MIS blocks.

To quantitatively assess the relative significance of various design parameters, we performed a comprehensive sensitivity analysis. Utilizing the control variable method, Figure 9 compares the impacts of WF, *d_h_*_-BN_, AlGaN doping concentration, Al Composition, interface trap densities, and SRH recombination on key performance metrics: *V*_th_, *I*_on_, and FOM.

The results clearly demonstrate that device performance is predominantly governed by four factors: WF, *d_h_*_-BN_, AlGaN doping concentration, and Al Composition. All these parameters exert a significant impact on FOM, which reflects their influence on the overall electrical behavior of the device. More specifically, the *V*_th_ is primarily modulated by the *d_h_*_-BN_, Al Composition, and WF, while exhibiting negligible sensitivity to the AlGaN doping concentration. Conversely, *I*_on_ shows a strong dependence on both AlGaN doping concentration and the *d_h_*_-BN_. These relationships collectively indicate that the device’s electrical characteristics are governed by the synergistic interplay between the thickness-dependent tunneling barrier of the *h*-BN insulating layer and the intrinsic properties of the AlGaN semiconductor layer.

In stark contrast to these dominant factors, the influence of interface traps and SRH recombination on device performance is virtually negligible. This finding provides compelling evidence that the van der Waals interface formed by the *h*-BN insulating layer possesses near-ideal characteristics, effectively suppressing the detrimental effects of both interface states and bulk recombination. Therefore, during device design and optimization, non-idealities of interfaces can be largely disregarded, allowing the focus to shift to core structural and material parameters.

**Figure 10:**
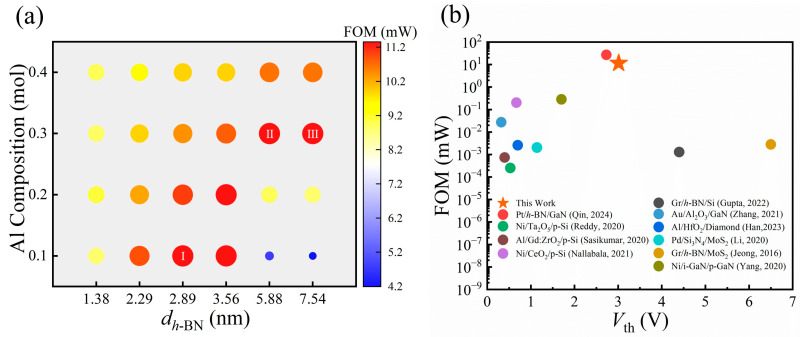
(**a**) Maximum FOM under different *d_h_*_-BN_ and Al Composition conditions; (**b**) comparison of the maximum FOM of this work with reported values for similar MIS blocks [18,20,21,29,30,31,32,33,36,62].

Comprehensive FOM analysis is presented for the parameter space of Al composition and *h*-BN thickness to establish the optimal design window for the MIS device. Figure 10a visualizes the maximum FOM achieved for each structural construction by co-optimizing the doping concentration and WF. The color and size of each data point are scaled to the FOM magnitude, and the top three optimal parameter combinations are explicitly highlighted. The analysis indicates that the optimal *h*-BN thickness required to maximize the FOM is strongly correlated with the Al Composition within the AlGaN layer. In particular, with increasing Al Composition, the FOM peak corresponds to a systematically greater *h*-BN thickness. For instance, an Al composition of 0.1 yields an optimal thickness of 2.89 nm (marked as “I”), whereas for a composition of 0.3, the optimum shifts to 5.88 nm (marked as “II” and “III”). This trend is further observed at an Al composition of 0.4, where the optimal window for the FOM becomes wider, with similar peak performance achieved at 5.88 nm and 7.54 nm. This trend is attributed to modulation of the AlGaN electron affinity and the device *R*_on_ by the Al composition. Increasing the Al composition in AlGaN lowers its electron affinity, which effectively mitigates the sharp increase in *R*_on_ with growing *h*-BN thickness. Consequently, for devices with a higher Al composition, a thicker *h*-BN insulating layer can be employed to achieve a higher *V*th without prematurely sacrificing the FOM due to excessive *R*_on_. Thus, the optimal design point shifts toward thicker *h*-BN barriers, which offers greater flexibility for high-voltage applications. In this study, a maximum FOM of 11.52 mW was achieved by systematically varying key parameters, including Al composition, the thickness of the *h*-BN layer, and WF. A comparative analysis of the maximum FOM between this work and previously reported values for similar MIS configurations is presented in Figure 10b [18,20,21,29,30,31,32,33,36,62]. This comparison is intended to establish a theoretical performance benchmark, reveal the potential application of this novel device structure in high-power electronics, and provide a quantifiable performance target for future experimental validation.

**Figure 11:**
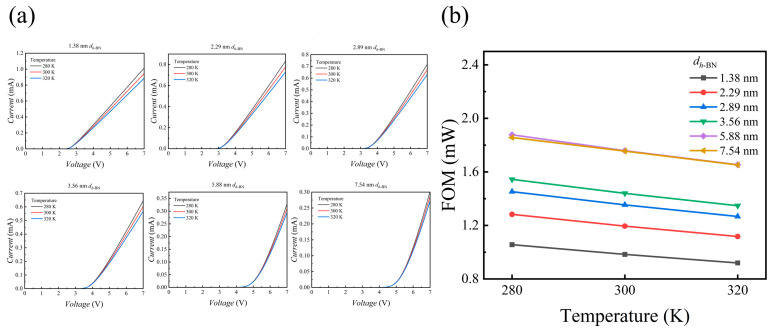
(**a**) Simulated *I–V* characteristics of devices with different *h*-BN thicknesses near room temperature, showing the device response as temperature varies from 280 K to 320 K in 20 K increments; (**b**) FOM as a function of temperature for MIS devices with various *h*-BN thicknesses. The plot illustrates the trend of performance degradation with increasing temperature.

Temperature dependence is a critical aspect for evaluating the practical potential and reliability of MIS devices. To assess the thermal characteristics of the AlGaN/*h*-BN MIS devices in this study, we analyzed their electrical performance near room temperature (280 K to 320 K) for various *h*-BN thicknesses, with an Al composition of 0.2 and a doping concentration of 1 × 10^16^ cm^−3^. As shown in Figure 11a, the device exhibits a counter-intuitive negative temperature coefficient.

This negative temperature coefficient is a distinct signature of an ideal transport regime where intrinsic material properties supersede non-ideal effects. This regime is enabled by the high-quality *h*-BN tunneling barrier, which facilitates temperature-independent F–N tunneling while suppressing temperature-sensitive leakage paths like trap-assisted tunneling [43,44]. With leakage paths suppressed, the thermal response is instead governed by enhanced phonon scattering in the AlGaN layer at higher temperatures. This scattering degrades carrier mobility, increasing the bulk resistance and lowering the electric field across the *h*-BN barrier, which ultimately suppresses the F–N current. Correspondingly, the device’s FOM exhibits a clear temperature dependence. As described in Figure 11b, the FOM for all devices decreases with rising temperature. Furthermore, the FOM trends for different *h*-BN thicknesses remain consistent across the tested temperature range. This consistency indicates that while absolute performance is temperature-sensitive, the design guidelines derived from our structural optimization are robust within the operational temperature window. This entire thermal behavior, theoretically investigated under the condition of an ideal interface formed by *h*-BN, establishes a fundamental performance benchmark dominated by intrinsic physics, offering a crucial theoretical reference for device fabrication and performance evaluation.

## 4. Conclusions

This work presents a systematic investigation into how key material and structural parameters influence the electrical characteristics of gold/*h*-BN/AlGaN MIS structures. The study reveals that increasing the Al Composition in the AlGaN layer gives rise to two competing physical phenomena. While it reduces the tunneling barrier height to enhance carrier transport, it concurrently increases the semiconductor bulk resistance, deteriorating the on-state performance. The dominance of these mechanisms is strongly governed by the thickness of the *h*-BN insulator. For devices with thinner *h*-BN layers, tunneling is facile, making the increased AlGaN bulk resistance the primary performance-limiting factor. In contrast, for those with thicker *h*-BN layers, tunneling is significantly impeded, and the reduction in the tunneling barrier height emerges as the key factor. This intricate interplay underscores a synergistic relationship between Al Composition and *h*-BN thickness.

Furthermore, this research clarifies the regulatory roles of other parameters: the doping concentration of AlGaN primarily determines the device’s on-resistance, whereas an increase in the metal WF elevates the interface barrier, leading to a simultaneous rise in both Vth and Ron. Notably, the nearly ideal van der Waals interface formed between *h*-BN and AlGaN effectively suppresses the influence of non-ideal interface effects on device performance. This finding is in excellent agreement with previous experimental results and highlights the unique advantage of *h*-BN as an insulating layer.

By analyzing the maximum achievable FOM across different combinations of Al composition and *h*-BN thickness, this study identifies a clear optimization pathway: to maximize the FOM, the optimal *h*-BN thickness must systematically increase with the Al composition in the AlGaN layer. The robustness of these design guidelines is further validated by the consistent performance trends observed across the operational temperature window. This principle provides quantitative design criteria for understanding and co-optimizing material and structural parameters, thereby laying a solid theoretical foundation for the future development of high-performance AlGaN/*h*-BN MIS electronic devices.

## Figures and Tables

**Table 1:** A comparative overview of MIS heterostructures employing conventional (3D) and 2D insulating materials.

	Conventional Insulator	2D Insulator
Conventional Semiconductor Material	Ni/Ta_2_O_5_/p-Si [32] Al/Gd:ZrO_2_/p-Si [33] Ni/CeO_2_/p-Si [31]	Gr/*h*-BN/Si [20]
Wide-Bandgap Semiconductor	Au/Al_2_O_3_/GaN [21] Al/HfO_2_/Diamond [29]	Pt/*h*-BN/GaN [18] Gold/*h*-BN/AlGaN (This work)
2D Semiconductor Material	Pd/Si_3_N_4_/MoS_2_ [30]	Gr/*h*-BN/MoS_2_ [36]

**Table 2:** Comparison of interfacial and transport properties in AlGaN MIS structures with *h*-BN (2D) versus Al_2_O_3_ (conventional) insulators.

Comparison Aspect	AlGaN/*h*-BN/Metal	AlGaN/Al_2_O_3_/Metal
Interfacial Bonding Mechanism	Van der Waals Physisorption [38]	Chemical Bond Formation [42]
Interface Trap Formation	Inherently Suppressed [38]	Inherently Present & Requires Engineering [42]
Dominant Carrier Transport	Fowler–Nordheim Tunneling	Hybrid of Direct Tunneling and Trap-Assisted Tunneling [42]
Thermal Management	Efficient In-Plane Heat Conduction [45]	Conventional Thermal Resistance [45]

**Table 3:** Fundamental physical parameters for AlGaN material used in this simulation.

Physical Phenomenon	Models	Parameters	GaN	AlN
BandGap	Temperature dependent bandgap model	Reference Bandgap (E_g0_) [eV]	3.53 [47]	6.23 [48]
		Reference Electron Affinity (Chi_0_) [eV]	4.1 [47]	0.6 [49,50]
		Alpha [eV/K]	9.09 × 10^−4^ [47]	1.79 × 10^−3^ [48]
		Beta [K]	830 [47]	1460 [48]
Mobility	Masetti Model	*μ*_const_ [cm^2^/Vs]	1800; 20 [48]	300; 14 [48]
		*γ_μ_*_max_	1; 2.1 [48]	1; 2.1 [48]
		*μ*_min1_ [cm^2^/Vs]	85; 33 [48]	20; 11 [48]
		*μ*_min2_ [cm^2^/Vs]	75; 0 [48]	65; 0 [48]
		*μ*_1_ [cm^2^/Vs]	50; 20 [48]	20; 10 [48]
		*P*_c_ [cm^−3^]	6.5 × 10^15^; 5 × 10^15^ [48]	8 × 10^17^; 5 × 10^18^ [48]
		*C*_r_ [cm^−3^]	9.5 × 10^16^; 8 × 10^16^ [48]	7 × 10^16^; 8 × 10^17^ [48]
		*C*_s_ [cm^−3^]	7.2 × 10^19^; 8 × 10^20^ [48]	5.2 × 10^17^; 8 × 10^18^ [48]
		α	0.55; 0.55 [48]	0.88; 1.05 [48]
		β	0.75; 0.7 [48]	0.75; 0.75 [48]
Recombination	SRH Recombination	Electron Lifetime (τ*_n_*); Hole Lifetime (τ*_p_*) [sec]	0.7 × 10^−9^; 2 × 10^−9^ [47]	1 × 10^−9^ [48]
Other basic Parameters of GaN and AlN		Dielectric Constant	8.9 [47]	8.5 [48]
		Electron Affinity [eV]	4.1 [47]	0.6 [50]
		Effective Electron mass [m_0_]	0.2 [47]	0.4 [51]
		Effective Conduction Band Density of states Nc [cm^−3^]	2.3 × 10^18^ [47]	6.3 × 10^18^
		Effective Hole mass [m_0_]	1.25 [47]	7.26 [52]
		Effective Valence Band Density of states Nv [cm^−3^]	3.5 × 10^19^ [47]	4.8 × 10^20^ [48]

## Data Availability

The original contributions presented in this study are included in the article. Further inquiries can be directed at the corresponding author.

## Abbreviations

The following abbreviations are used in this manuscript:

F–N	Fowler–Nordheim
MIS	Metal/insulator/semiconductor
MIM	Metal/insulator/metal
MOCVD	Metal–organic chemical vapor deposition
SRH	Shockley–Read–Hall
Dit	The interface trap density
*V*th	The threshold voltage
*I*_on_	The on-state current
$R_{net}^{SRH}$	The SRH recombination rate
*E*_trap_	The energy offset between the intrinsic Fermi level and the trap level
*J*_F-N_	The tunneling current density
*d**_h_*_-BN_	The thickness of *h*-BN
*I*–*V*	The current–voltage
*R*_on_	The on-state resistance
*F*_ins_	The magnitudes of the electric field in *h*-BN
WF	Work function
FOM	The power figure-of-merit
